# Sequential Donation: Evaluating Safety and Quality of Life of Living Donors

**DOI:** 10.1111/ctr.70609

**Published:** 2026-07-02

**Authors:** Jennifer L. Steel, Amit Tevar, Swaytha Ganesh, Tadas Trakumas, Marko Heuer, Maya L. Maurer, Emily Tillman, Sarah Meketa, Deborah Mauer, Rekha Ramanathan, Lindsay Weslow, Andrea DiMartini, Shanthi Mayadam, Alexandra Mullen, Ana Yandrich, Cramer J. Kallem, Puneet Sood, Michele Molineri, Shivani Jajoo, Vikraman Gunabushanam, Abhinav Humar

**Affiliations:** ^1^ Department of Surgery University of Pittsburgh Pittsburgh Pennsylvania USA

**Keywords:** complications, health behaviors, living kidney donor, living liver donor, quality of life, sequential donor, unplanned health care utilization

## Abstract

One of the most persistent problems in transplantation in the U.S. is the demand for organs outweighs the supply. Sequential living donation may be one strategy to address this problem, however a reluctance to perform these surgeries exists secondary to the lack of research on the medical outcomes and quality of life of sequential post‐donation. A medical record review of 34 sequential liver and kidney living donors and matched controls (by sex, age within 7 years, timing of surgery, and last organ donated) was performed. No significant difference between groups were observed on sociodemographic characteristics with the exception of age [*p* = 0.030] and marital status [*p* = 0.010] whereas matched controls were older and more likely to be married. One sequential donor started a chain for both organs which resulted in 7 transplant candidates receiving an organ. No statistically significant differences were observed in the number of complications [*p* = 0.081], emergency room visits [*p* = 0.573], 30‐day [*p* = 0.573], 90‐day readmissions [*p* < 0.999], or physical [*p* = 0.09] or mental health domains of quality of life [*p* = 0.78] at six or more months post‐donation. Sequential donors returned to tobacco use more often than controls [*p* = 0.03] and matched controls were more likely to gain weight [*p* = 0.02] post donation. While further research is warranted, this study provides early evidence of the short‐term safety of sequential donation.

**Abbreviations**
eGFR: estimated Glomerular Filtration Rate
IBM: International Business Machine
MOS SF: medical outcome survey short form
NLDAC: National Living Donor Assistance Center
PHQ: patient health questionnaire
SPSS: Statistical Package for the Social Sciences

## 1 Introduction

One of the most persistent problems in transplantation is that the demand for organs outweighs the supply in the U.S. and as a result increases the risk of morbidity and mortality for those awaiting a liver or kidney transplant [1, 2]. While the number of available organs has increased by nearly 10% in the past year, there were still over 100 000 people waiting for an organ and approximately 24 people die each day waiting for a kidney or liver [3]. Several strategies to reduce the gap between the demand and supply have been implemented, including living donation.

While living kidney donation has been performed for decades, only a limited number of transplant centers perform living liver donor transplants. Recipients who receive a living liver donor organ have a shorter wait time and a lower risk of graft rejection and improved survival compared to transplants from deceased liver donors [4].

The motivations of living donors have been studied and personal loss, the opportunity to help someone in need, and a positive interaction with transplant recipients were all reported as common reasons to be a directed and non‐directed donor [5, 6, 7]. Many non‐directed living liver donors also reported prior altruistic acts, such as blood donation, volunteering, and/or being a kidney donor [8].

Few centers in the world consider performing sequential donor surgeries which often include at least one directed or non‐directed donation. For the purposes of this paper, a sequential donor is defined as an individual who donates a kidney and part of their liver sequentially, with at least one year between donations. While some countries allow a donation of a kidney and part of the liver simultaneously, this has not been performed in the U.S. to our knowledge [9, 10].

Many transplant surgeons are reluctant to perform directed or non‐directed donor surgeries secondary to the possibility of poor outcomes and the effect it can have on the entire center and the psychological sequelae of the donor if the recipient has complications or passes away. However, prior research has shown that these donors have improved quality of life and lasting improvements in self‐esteem, even if the donor or the transplant recipient experienced complications [11].

Limited research exists on sequential donation and, of the two studies that have been conducted, the focus has been on medical complications. Of the few studies that have been performed regarding sequential donation, investigators reported that a single liver complication was secondary to the prior kidney donation [10]. In general, there was no indication that the complication rates increased after the first organ donation however the samples studied have been small and limited in number [9, 10].

In the present study, we will examine the differences between sequential donors and matched controls on medical outcomes (e.g., complication rates, readmissions), psychosocial outcomes (e.g., depression and quality of life), health related behaviors (e.g., tobacco and alcohol use), as well as economic consequences (e.g., out of pocket expenses, insurance status). Matched controls in this study were biologically or emotionally related kidney and liver donors. Matching was based on the sequential donors’ sex, age within 7 years, timing of surgery (within 6 month), and last organ donated. Biologically and emotionally related donors are considered by all transplant centers to be acceptable donor candidates, if they meet the criteria for that specific transplant center.

## 2 Methods

### 2.1 Design

The study was a retrospective chart review case‐control design that included matching living donors who donated a kidney and part of their liver sequentially (case) with matched controls who were emotionally or biologically related to their recipients and donated either a kidney or part of a liver (controls).

### 2.2 Participants

A total of 34 consecutive sequential living donors and 34 matched controls were included in the study. The living donors were evaluated at a large transplant center that evaluates and performs living kidney and liver donor surgeries. The sequential donors and matched controls met the same eligibility criteria depending on the organ and were evaluated by a multidisciplinary team as with all donors who present for living donation. The Independent Living Donor Advocate met with all donors to assure there was no pressure or coercion, including from the medical teams. The only difference in was that sequential donors, who had donated a kidney first, were permitted to be evaluated for liver donation if they had an eGFR ≥60. All donors were required to have at least a year between donations.

All participants underwent at least one surgery at the index transplant center. If one of the surgeries was performed at another center, the follow up care of the donor was performed by that center. The sequential donor was matched with a control donor who was emotionally or biologically related to their transplant candidate and had either a kidney or part of the liver donated but not both organs. The match was based on the organ last donated for the case (e.g., if the last organ donated by the sequential donor was a liver, they were matched with a liver donor control, if the last organ donated by the case was a kidney, the matched control donor was an emotionally or biologically related kidney donor). Cases and controls were also matched according to age (within 7 years), biological sex at birth (male/female), and duration between surgeries of the case and control (within 7 months). For the post‐operative interviews, all donors were provided a description of the study and verbal consent was obtained prior to the interview.

### 2.3 Assessment or Evaluation of Living Donors

Pre‐ and post‐donation chart reviews included information from the electronic medical record that included evaluations by the nephrologists, hepatologists, surgeons, social workers, psychiatrist, and independent living donor advocates. Pre‐ and post‐donation laboratory data was collected as well as post‐surgery complications, length of stay, emergency room visits, and readmissions from the electronic medical record. Post‐surgery evaluations by the social workers and independent living donor advocates were collected from the medical record. The ILDA also performed evaluations beginning 6‐months and up to 10 years after the surgery to assess medical, psychosocial and economic outcomes of donors for the purposes of this study. During these interviews, standardized instruments were also used to collect information on altruism, depressive symptoms, and quality of life including:

**Altruism Scale**: The Altruism Scale is a 20‐item scale designed to measure altruistic tendency by assessing the frequency an individual engages in altruistic acts that are directed primarily toward strangers. Participants answer on a 5‐point scale ranging from Never (0) to Very Often (4). The scale has demonstrated good construct validity [12].

**Patient Health Questionnaire‐2**: The Patient Health Questionnaire (PHQ)‐2 is a 2 item self‐report scale that assesses frequency and severity of depressive symptoms. The scale has a sensitivity and specificity of 78%–92% and 91%–94%, respectively [13].

**Rand Medical Outcomes Short‐Form 36**: The Medical Outcomes Short‐Form (MOS SF) 36 is a 36 item self‐report measure of health. It assesses overall health related quality of life and has been widely used across populations with chronic illness. The MOS SF‐36 has a high level of reliability (Cronbach alpha = 0.90) and excellent convergent validity [14].

### 2.4 Procedure

Institutional Review Board approval for the retrospective chart review was obtained prior to the commencement of the study. The medical chart review included both pre‐ and post‐donation evaluations by the transplant team (e.g., surgeon, social work, psychiatry) and the independent living donor advocate team.

### 2.5 Data Analysis

Data analyses was performed using International Business Machine(IBM) Statistical Package for the Social Sciences (SPSS) version 28 (Chicago, IL). Descriptive statistics were performed to provide sociodemographic characteristics. Analysis of variance was performed to test differences between the two cohorts of patients (sequential donors and matched controls) with continuous, normally distributed outcomes and chi‐square and Fisher's exact analyses were performed with categorical outcomes. Non‐parametric tests were performed to assess differences between non‐normally distributed outcomes but age was not adjusted. The *p*‐value of <0.05 was set as the criteria for statistical significance.

## 3 Results

Sociodemographic characteristics of the sequential and matched control donors can be found in Table 1.

**TABLE 1:** Characteristics of sequential donors and matched controls.

	Sequential donors (*n* = 34)	Matched controls (*n* = 34)	Between group *p*‐value
Male (*n*, %)	15 (44)	15 (44)	0.91
Age (mean, SD)	38 (0.2)	43 (7.8)	0.03
Caucasian (*n*, %)	32 (94)	34 (100)	0.37
College education (*n*, %)	31 (91)	21 (62)	0.27
Occupation in helping field (*n*,%)	10 (29)	4(12)	0.82
Married or cohabitating with partner (*n*, %)	11 (32)	23 (68)	0.01
First donation at another medical center (*n*, %)	7 (21)	—	—
**Health behaviors and psychosocial history**			
Tobacco use at evaluation (*n*, %)	1 (3)	5 (15)	0.22
Past tobacco use (*n*, %)	0 (0)	8 (24)	0.34
Alcohol use at evaluation (*n*, %)	28 (82)	23 (68)	0.22
Drug use at evaluation (*n*, %)	1 (3)	1 (3)	0.98
Past drug use (*n*, %)	4 (12)	10 (29)	0.06
History of depression at evaluation (*n*, %)	8 (24)	5 (15)	0.39
History anxiety at evaluation (*n*, %)	5 (15)	10 (29)	0.13
Psychopharmacology at evaluation (*n*, %)	10 (29)	9 (26)	0.85
History of inpatient psychiatric hospitalization (*n*, %)	2 (6)	2 (6)	<0.999
History of outpatient therapy (*n*, %)	12 (35)	9 (26)	0.48
Family history of psychiatric disorders (*n*, %)	8 (24)	8 (24)	<0.999

**FIGURE 1:**
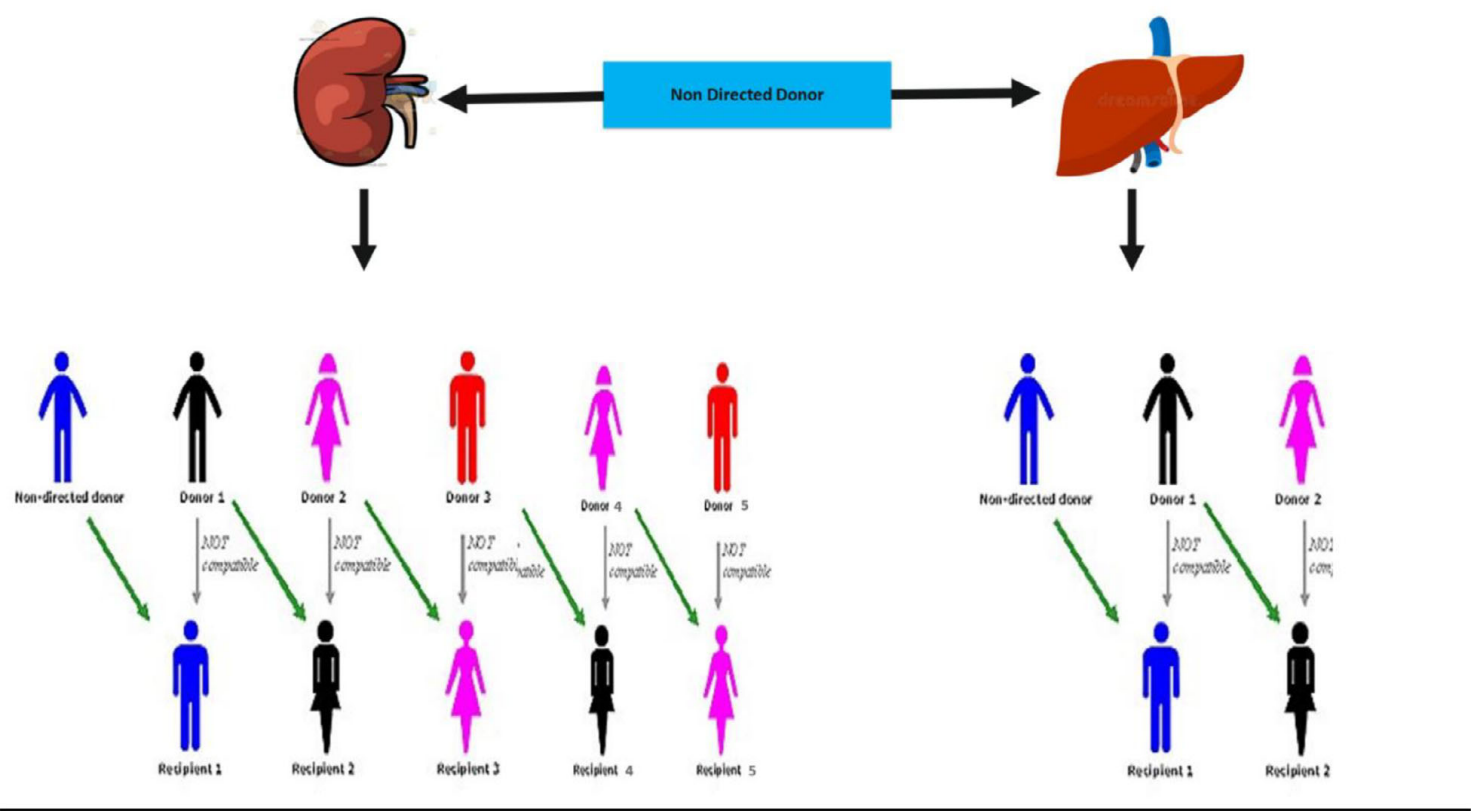
Sequential living donor who participated in a kidney and liver donor chain.

No significant differences between groups was observed prior to surgery with the exception of age and marital status. Mean age of the sequential donors was 38 (S.D. = 0.2) and 43 (SD = 7.8) for matched controls. Of the matched controls, 68% were married versus only 32% of the sequential donors. The sequential donors often donated to two separate recipients (liver and kidney), however one living donor donated a kidney and liver that started a chain and resulted in two people receiving a liver and five people receiving a kidney. See Figure 1.

Table 2 provides information regarding the organ donation order, relationship to the recipients, whether the first organ donation was performed at the index center, the interval between organ donations, and post‐operative timing of interview. A total of 24 donors had both surgeries performed at the index center and 24 donors (not the same donors) donated a kidney first. The mean interval between surgeries was 4.5 years (SD = 5.0) and the mean time to interview after the second surgery was 2.2 years (SD = 1.8).

**TABLE 2:** Characteristics of sequential donors.

Sequential donor	First organ donated	Relationship with recipients (first/second)	First organ performed at index center (Yes/No)	Interval between surgeries (years)	Post‐operative timing of interview (years)
1	Kidney	Non directed Non directed	No	1	3
2	Kidney	Non directed Non directed	No	11	3
3	Liver	Non directed Directed	Yes	1	2
4	Kidney	Non directed Non directed	No	4	1
5	Kidney	Friend Non directed	Yes	2	3
6	Kidney	Father Non‐directed	Yes	1	2
7	Kidney	Non directed Non directed	Yes	7	1
8	Kidney	Sibling Sibling	No	26	2
9	Kidney	Non directed Non directed	No	10	10
10	Kidney	Directed Directed	No	3	2
11	Liver	Non directed Non directed	Yes	2	2
12	Kidney	Non directed Friend	No	1	1
13	Kidney	Non directed Directed	No	4	1
14	Liver	Sibling Sibling	Yes	2	2
15	Liver	Non directed Non directed	Yes	1	3
16	Liver	Friend Non directed	Yes	2	2
17	Liver	Father Non directed	Yes	6	3
18	Kidney	Non directed Non directed	Yes	4	1
19	Kidney	Friend Non directed	Yes	3	3
20	Liver	Non directed Non directed	Yes	2	2
21	Liver	Directed Non directed	Yes	1	2
22	Kidney	Cousin Sibling	Yes	14	2
23	Kidney	Sibling Non directed	Yes	1	1
24	Kidney	Friend Non directed	Yes	3	1
25	Kidney	Non directed Directed	Yes	7	7
26	Kidney	Non directed Non directed	Yes	9	1
27	Kidney	Directed Directed	Yes	3	4
28	Kidney	Non directed Non directed	Yes	1	1
29	Kidney	Non directed Non directed	Yes	3	2
30	Liver	Directed Directed	Yes	5	1
31	Liver	Non directed Non directed	Yes	4	1
32	Kidney	Non directed Non directed	No	6	1
33	Kidney	Non directed Non directed	No	1	1
34	Kidney	Non directed Non directed	Yes	2	1

No statistically significant differences were observed with the sequential donors experiencing a greater number of complications [Fisher's Exact χ^2^ = 3.203, *p* = 0.082] including by Clavien Dindo Grade [χ^2^ = 3.667 *p* = 0.300] or emergency room visits [F(1,66) = 0.310, *p* = 0.579], 30‐day readmissions [Fisher's Exact Test = 1.001, *p* = 0.318] or 90‐day readmissions [Fisher's Exact χ^2^<0.999, *p* < 0.999], see Table 3.

**TABLE 3:** Post donation complications and unplanned health care utilization.

	Sequential donor (*n*, %)	Matched control (*n*, %)	*p*‐value
**Clavien Dindo category (number of patients)**	—	—	0.300
Clavien Dindo I	1	0	
Clavien Dindo II	3	1	
Clavien Dindo III	6	3	
Clavien Dindo IV	0	0	
**Complications type (Clavien Dindo classification)**			—
Abscess (II)	1	0	—
Small bowel adhesion requiring surgery (III)	2	1	—
Umbilical hernia after kidney (III)	2	0	—
C diff infection (II)	1	0	—
Ileus and pulmonary embolism (III)	1	0	—
Difficulties urinating after liver surgery (I)	1	0	—
Neuropraxia (III and II)	1	0	—
Wound Dehiscence (II)	0	1	—
Pneumonia (II)	0	1	—
Incisional hernia (III)	2	2	—
**Emergency room visits**	2	1	0.573
**30‐day readmissions**	3	1	0.317
**90‐day readmissions**	0	0	<0.999

Laboratory values were significantly different between cases and controls with creatinine and eGFR being significantly different between the sequential donors and matched controls. See Table 4. Post hoc analyses also demonstrated significant differences in eGFR and creatinine between those sequential donors who donated a kidney versus part of their liver first. See Table 5.

**TABLE 4:** Laboratory results pre‐ and post‐donation: Sequential versus matched control donors.

Pre‐donation	Sequential donor	Matched control	*p*‐value
eGFR (ML/min/1.73m^2^, median, IQR)	76 (20.8)	90 (27.5)	<0.001
Creatinine (mg/dL median, IQR)	1.0 (0.3)	0.9 (0.20)	0.001
Glucose (mg/dL, median, IQR)	88 (17.0)	91 (16.5)	0.748
Bilirubin (mg/dL, median, IQR)	0.6 (0.2)	0.6 (0.30)	0.974
ALT (U/L, median, IQR)	15.5 (7.8)	18 (17.0)	0.011
AST (U/L, median, IQR)	19.0 (7.3)	20 (10.5)	0.229
ALK phosphate (U/L, median, IQR)	56.5 (30.0)	58 (22.5)	0.984
Albumin (g/dL, median, IQR))	4.54 (0.26)	4.49 (0.29)	0.404
Systolic blood pressure (mm Hg, mean, SD)	122.4 (14.7)	123.9 (15.2)	0.677
Diastolic blood pressure (g/dL, mean, SD)	72.4 (9.1)	73 (11.7)	0.810
**Day of donation**			
eGFR (ML/min/1.73m^2^ median, IQR)	74 (25)	95 ((16.8)	0.003
Creatinine (mg/dL median, IQR)	1.1 ().4)	0.9 (0.18)	0.012
Glucose (mg/dL, median, IQR)	142 (44)	134 (16.5)	0.292
Bilirubin (mg/dL, median, IQR)	1.40 (0.67)	1.40 (0.35)	0.681
ALT (U/L, median, IQR)	260 (123.0)	266 ((159.5)	0.749
AST (U/L, median, IQR)	242.5 (120.8)	244 (139.0)	0.250
ALK phosphate (U/L, median, IQR)	42.5 (20.0)	46 (20.0)	0.889
Albumin (g/dL, median, IQR)	—	—	—
Systolic blood pressure (g/dL, mean, SD)	122.7 (16.7)	124 (15.9)	0.755
Diastolic blood pressure (g/dL, mean, SD)	76.6 (9.8)	75.8 (8.7)	0.746
**6‐months post donation**			
eGFR (ML/min/1.73m^2^, median, IQR)	68 (16.0)	92 (41.0)	0.003
Creatinine (mg/dL median, IQR)	1.1 (0.20)	0.95 (0.3)	0.001
Glucose mg/dL, (median, IQR)	89 (11.5)	89.5 (11.8)	0.901
Bilirubin (mg/dL, median, IQR)	0.6 (0.20)	0.65 (0.5)	0.691
ALT (U/L, median, IQR)	16 (8.5)	22 (15.3)	0.008
AST (U/L, median, IQR)	22.5 (9.5)	23.5 11.3)	0.595
ALK Phosphate (U/L, median, IQR)	64 (32.2)	71.5 ((42)	0.602
Albumin (g/dL, median, IQR)	4.23 (0.24)	4.27 (0.45)	0.724
Systolic blood pressure (g/dl, mean, SD)	125.4 (13.1)	126.2 (20.5)	0.891
Diastolic blood pressure (g/dl, mean, SD)	74.9 (7.9)	74.8 (14.7)	0.977
**12‐months**			
eGFR (ML/min/1.73m^2^, median, IQR)	67 (19.0)	87 (29.5)	0.005
Creatinine (mg/dL median, IQR)	1.1 (0.30)	1.0 (0.3)	0.008
Glucose (mg/dL, median, IQR)	88 (11.5)	91.5 3.75)	0.137
Bilirubin (mg/dL, median, IQR)	0.6 (0.28)	0.6 (0.43)	0.697
ALT (U/L, median, IQR)	19 (11.0)	21.5 (12.0)	0.165
AST (U/L, median, IQR)	22.5 (7.5)	22 (13.0)	0.783
ALK Phosphate (U/L, median, IQR)	68.5 (21.8)	62 20.5)	0.481
Albumin (g/dL, median, IQR)	4.35 (0.23)	4.38 (0.33)	0.673
Systolic blood pressure (g/dl, mean, SD)	122.4 (12.4)	122.8 (13.9)	0.921
Diastolic blood pressure (g/dl, mean, SD)	71.5 (11.6)	73.0 (9.8)	0.640

**TABLE 5:** Laboratory results pre‐ and post‐donation: Donors who donated a kidney versus liver first.

Pre‐donation	Donated kidney first (*n* = 24)	Donated liver first (*n* =10)	*p*‐value
eGFR (ML/min/1.73m^2^, median, IQR)	72 (15.5)	92.5 (22.8)	<0.001
Creatinine (mg/dL median, IQR)	1.1 (0.3)	0.8 (0.15)	0.002
**Day of donation**			
eGFR (ML/min/1.73m^2^ median, IQR)	77 (23)	54 (15.5)	0.002
Creatinine (mg/dL median, IQR)	1.05 (0.5)	1.2 (0.3)	<0.001
**6‐months post donation**			
eGFR (ML/min/1.73m^2^, median, IQR)	71 (14.5)	60.5 (10)	0.003
Creatinine (mg/dL median, IQR)	1.1 (0.23)	1.1 (0.11)	0.742
**12‐months**			
eGFR (ML/min/1.73m^2^, median, IQR)	67.5 (17)	57 (12)	0.028
Creatinine (mg/dL median, IQR)	1.1 (0.3)	1.12 (0.28)	0.945

**FIGURE 2:**
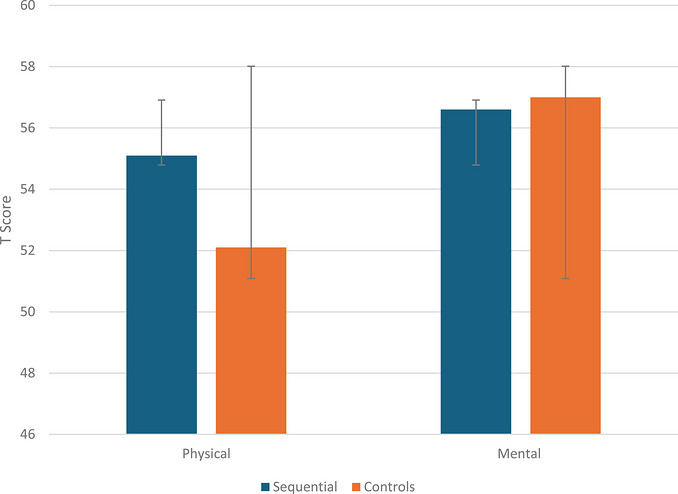
Rand Medical Outcome Study Short Form 12 of the sequential donors and matched controls.

Moreover, no significant differences were found between groups on the Rand Medical Short Form‐36 physical [F(1,43) = 3.1, *p* = 0.09] or mental domain [F(1,43) = 0.8, *p* = 0.78] six or more months post‐donation, see Figure 2.

Significant differences were observed on the altruism questionnaire with sequential donors reporting a greater number of altruistic acts (Mean = 66.2, SD = 11.1) than the matched controls (Mean = 55, SD = 6.0; F(1,18) = 7.2, *p* = 0.02). No significant differences were reported by donors with regard to depressive symptoms measured by the Patient Health Questionnaire‐9 post donation (F(1,43) = 1.3, *p = *0.27).

Significant differences between cases and controls were observed with regard to return to tobacco use [χ^2^ = 4.6, *p* = 0.03] and changes in weight [χ^2^ = 6.0, *p* = 0.02]. Two sequential donors and 1 matched control donor returned to tobacco use post donation while 3 matched control donors did not return to tobacco use. The changes in weight included 4 sequential and 5 matched controls losing weight (5–10 pounds) and 4 sequential donors and 8 matched controls gaining weight (5–10 pounds) six or more months after one or both surgeries.

No significant differences were observed post‐donation with regard to changes in alcohol use [χ^2^ = 1.4, *p* = 0.24], drug use [χ^2^ = 0.44, *p* = 0.51], physical activity [χ^2^ = 0.12, *p* = 0.72], or preventative care [χ^2^ = 1.7, *p* = 0.19]. No changes in health [χ^2^ = 0.70, *p* = 0.40], disability [χ^2^ = 0.01, *p* = 0.99], or life insurance [χ^2^ = 0.70, *p* = 0.40] were observed between cases and controls. We also did not observe any differences in out of pocket expense for medications [χ^2^ = 0.015, *p* = 0.90], lost wages [c^2^ = 0.90, *p* = 0.34], child or pet care [χ^2^ = 1.43, *p* = 0.23], transportation [χ^2^ = 0.81, *p* = 0.37], or lodging [χ^2^ = 0.003, *p* = 0.96] despite the sequential donors donating twice. No significant differences were observed with regard to the type of time taken away from work including vacation time [χ^2^ = 0.04, *p* = 0.84], sick time [χ^2^ = 0.98, *p* = 0.32] Family Medical Leave Act type of leave [χ^2^ = 2.2, *p* = 0.14], short‐term disability [χ^2^ = 0.21, *p* = 0.65], or use of donor benefit from their employer [χ^2^ = 1.5, *p* = 0.22].

## 4 Discussion

This study provides early short‐term evidence that sequential donation does not result in greater medical, psychosocial, or financial morbidity or mortality when compared to biologically or emotionally related donors who donated a single organ, which is widely accepted in the U.S. Despite the sequential donors having two surgeries, statistically significant differences in rates of complications and types of complications were not observed. Similarly, unplanned health care utilization was not greater for the sequential donors despite having two surgeries. No complications were greater than Grade III. Moreover, differences between the two cohorts on measures of health‐related quality of life at six or more months after donation were not observed. This finding is consistent with prior research which also found that live organ donors’ quality of life was as good as the general population's [15]. Moreover, sequential donors also did not differ significantly in regard to depressive symptoms pre‐ or post‐donation when compared to matched controls.

While we observed that the sequential donors’ serum levels of creatinine was higher and estimated Glomerular Filtration Rate (eGFR) was lower atpre‐donation, 6‐ and 12‐months, the lower lab values were expected and aligns with prior research. Kidney donors tend to experience a 25%–30% decrease in kidney function after living donation and this was observed for both the sequential and living kidney donors [16]. Further, studies of kidney donation have shown that while kidney donors do have an increased risk of end stage kidney disease [17], the actual rate does not exceed the rate of the general population in the United States [18]. However, continued short‐ and long‐term surveillance of creatinine and eGFR of living donors by their primary care physician is recommended.

In this study, 24 of the 34 (71%) of the sequential donors had donated a kidney first resulting in lower overall kidney function pre‐donation. The index center required an eGFR of 60 or greater to proceed with living liver donation after kidney donation. Post hoc analyses results show significant differences between the sequential donors who donated a kidney first versus part of their liver however this was likely due to the small sample size. Absolute eGFR and creatinine were higher in those who donated a part of their liver first. Motivation for donating a second organ was primarily associated with the positive experience of donating the first organ, observing a significant improvement in health of their recipient, and the desire to help another person.

Sequential donors also reported greater levels of previous acts of “altruism” when compared to biological and emotionally related donors. This correlates with the increased frequency of sequential donors being employed in helping fields or having a close friend or family member affected by a chronic illness which consistent with prior literature [8]. Our findings mirror prior studies of non‐sequential, non‐directed donors which have found that donors had a history of helping others and saw the donation as an extension of their identity with many having a history of volunteering and other helping behaviors [19, 20]. Further, previous studies of those who are “altruistic” including live organ donors, found that they rated higher on measures of honesty‐humility when compared to those who may be less “altruistic,” suggesting altruism mirrors other prosocial behaviors [20].

We observed significant differences in weight after donation, which has been found in other studies [21]. Increased weight, albeit modest, was more common with the matched controls. While the weight gain was limited, ongoing surveillance by transplant and primary care teams is recommended to reduce the risk of developing weight related chronic diseases (e.g., high cholesterol, hypertension). Both cohorts of donors, but particularly sequential donors, who used tobacco prior to surgery, resumed use of tobacco products following donation surgery. Increased efforts for tobacco cessation are recommended for all donors by the transplant center and/or health care organization.

Financially, there were no significant differences between matched controls and sequential donors with regard to out‐of‐pocket expenses, even despite the sequential donors having two surgeries and recovery periods. However, due to the limited number of centers that allow for sequential donation, some donors may be required to travel further from home and stay away from home for prolonged periods, which results in increased out‐of‐pocket expenses and/or expenses reimbursed by the National Living Donor Assistance Center (NLDAC). As a result, with an increase in the number of centers willing to offer sequential living donor surgeries, the cost to donors and taxpayers, who contribute to the NLDAC, may be reduced.

While our study has many strengths including the first and largest sample size of sequential donors in which medical and psychosocial factors were examined, our study is limited by a small sample size, as there are still very few sequential donor surgeries performed in the United States. As of 2022, there were only 150 living sequential organ donors within the United States that had ever been performed [4]. Our study contains results only from a single transplant center. Both factors limit the generalizability of our results to other centers or geographic regions. The matching was determined a priori and due to the limited number of donors with all matching criteria, the age distribution between sequential donors and matched controls was significantly different despite our best efforts to match donors within 7 years which is a limitation of the study. This likely serves as an explanation for other significant differences between groups, such as marriage or cohabitation with a partner, which was more frequent within our matched controls, and is expected to be higher among older donors. While we gathered information from the patient as well as from the medical record, we only had self‐reported complications, ER visits, and readmissions from patients when the surgery was performed at another transplant center.

Recommendations from our study suggest that ongoing surveillance of kidney donors after their donation is recommended at least annually by their primary care physician after the two year surveillance has been performed by the transplant center. Both creatinine and Glomerular Filtration Rate would be critical to obtain at these visits to help identify potential reduced kidney function early, but this is true for both single live kidney donors as well as sequential donors. It does not appear that those donors who also gave a part of their liver had worse kidney function than those that donated a kidney alone. Lifestyle management is also recommended for living donors and especially sequential donors. Education regarding lifestyle management could start after donation since donors are followed closely for the first two years after donation [22]. Early intervention is critical to avoid long‐term health consequences including returning to tobacco use or weight gain after surgery.

Further, both within our study and prior research, some donors expressed increased stress surrounding donation due to the perceived, and actual, financial burden created by donating [23, 24]. The National Living Donor Assistance Center (NLDAC) was a significant contributing factor for many donors feeling comfortable from financial perspective to donate a single organ, or sequentially. Further work at decreasing the financial strain on donors may improve outcomes and improve general willingness to participate in live organ donation.

Based on the lack of differences in medical, psychosocial, or financial complications post‐donation between sequential donors and matched controls, it is worth considering expanding sequential donation across more centers in the U.S. Further research is recommended due to the limited sample size. A registry of sequential donors across transplant centers is recommended to better understand the unique contributions and potential long‐term risks for these donors.

With the appropriate evaluation and testing prior to donation, surgical expertise, appropriate post‐operative care, and surveillance of the donors; the findings of this study provide early evidence for the short‐term psychological and physical safety of sequential donation. However, future research is warranted with a larger sample of sequential donors and longer follow‐up.

## Funding

Clinical and Translation Sciences Institute UL1‐TR‐001857 for RedCap.

## Conflicts of Interest

The authors declare no conflicts of interest.

## Data Availability

The data that support the findings of this study are available on request from the corresponding author. The data are not publicly available due to privacy or ethical restrictions.
